# A Case of Spontaneous Isolated Celiac Artery Dissection with Pseudoaneurysm Formation

**DOI:** 10.7759/cureus.1616

**Published:** 2017-08-27

**Authors:** John Kim, Lamar H Moree, Michael J Muehlberger

**Affiliations:** 1 Student, UCF College of Medicine; 2 Surgery, Orlando Regional Medical Center; 3 Vascular Specialists of Central Florida, Orlando Regional Medical Center

**Keywords:** vascular disease, celiac artery, dissection, pseudoaneurysm, spontaneous isolated celiac artery dissection, aneurysm, endovascular outcomes, treatment management, conservative treatment

## Abstract

Spontaneous isolated celiac artery dissection is a rare disease and patients without evidence of significant complications often resolve with medical therapy alone; however, the extension of the dissection can lead to more serious complications including aneurysmal dilatation, complete occlusion, and rupture of a visceral artery. In these patients, optimal management has not yet been clearly defined and treatment primarily depends on clinical presentation and lesions identified on imaging studies. This case report demonstrates the conservative management of spontaneous celiac artery dissection.

A 49-year-old male presented to our emergency department with acute and persistent abdominal pain. A contrast-enhanced computed tomogram (CT) of abdomen showed a pseudoaneurysm involving the ostium of the celiac artery with focal dissection, with no evidence of thrombosis or infarction to the visceral organs. Ultrasound studies demonstrated a prominent but patent celiac artery with adequate distal perfusion. Therefore, conservative medical management with antiplatelet therapy was initiated in our patient. Follow-up repeat ultrasound three weeks following discharge showed no evidence of dissection flap, stenosis, thrombosis or increase in the size of the aneurysm.

This case report demonstrates that conservative medical management with antiplatelet therapy can be sufficient in treating spontaneous isolated celiac artery dissection with pseudoaneurysm formation. We suggest endovascular or surgical intervention should be reserved for patients who present with hemodynamic instability, or other serious complications, such as aneurysm rupture or visceral infarction.

## Introduction

Spontaneous isolated celiac artery dissection is a rare disease process. Although a minority of patients may present with sudden onset abdominal pain, most reported cases of celiac artery dissection are asymptomatic and found incidentally on computed tomography (CT) imaging. Since such few cases have been reported in the literature, the management of patients with this condition has not yet been clearly defined. Conservative medical treatment, endovascular procedures, and surgical revascularization are the primary treatment options for managing celiac artery dissections. Prior literature suggests that surgical or endovascular procedures are recommended in patients who have evidence of visceral organ ischemia, aneurysmal degeneration, or continued propagation of the dissection, whereas other stable patients with no complications can be managed conservatively with antiplatelet therapy and blood pressure control. This case report outlines the decision to pursue conservative medical management of a 49-year-old male who presented with acute abdominal pain and was diagnosed with spontaneous isolated celiac artery dissection with pseudoaneurysm formation.

## Case presentation

A 49-year-old male with no significant past medical history presented to our emergency department with a 5-day history of sudden-onset and persistent abdominal pain. His pain began acutely following a strenuous bike ride and had not improved throughout the week. He described constant epigastric pain rated 7/10 with radiation into his back that worsened with eating. He denied nausea, vomiting, constipation, or diarrhea. He also denied chest pain, palpitations, shortness of breath, or any recent history of trauma. His past surgical history was only significant for a remote knee arthroscopy. Upon initial examination, he was afebrile and alert with the blood pressure of 139/95. His cardiopulmonary examination was normal. His abdomen was tender upon palpation of the epigastric region; however, there was no notable distension, no pulsatile abdominal mass, and his abdomen was soft with positive bowel sounds. Initial laboratory tests including troponin levels, complete blood count, and renal function tests were within normal limits.

**Figure 1:**
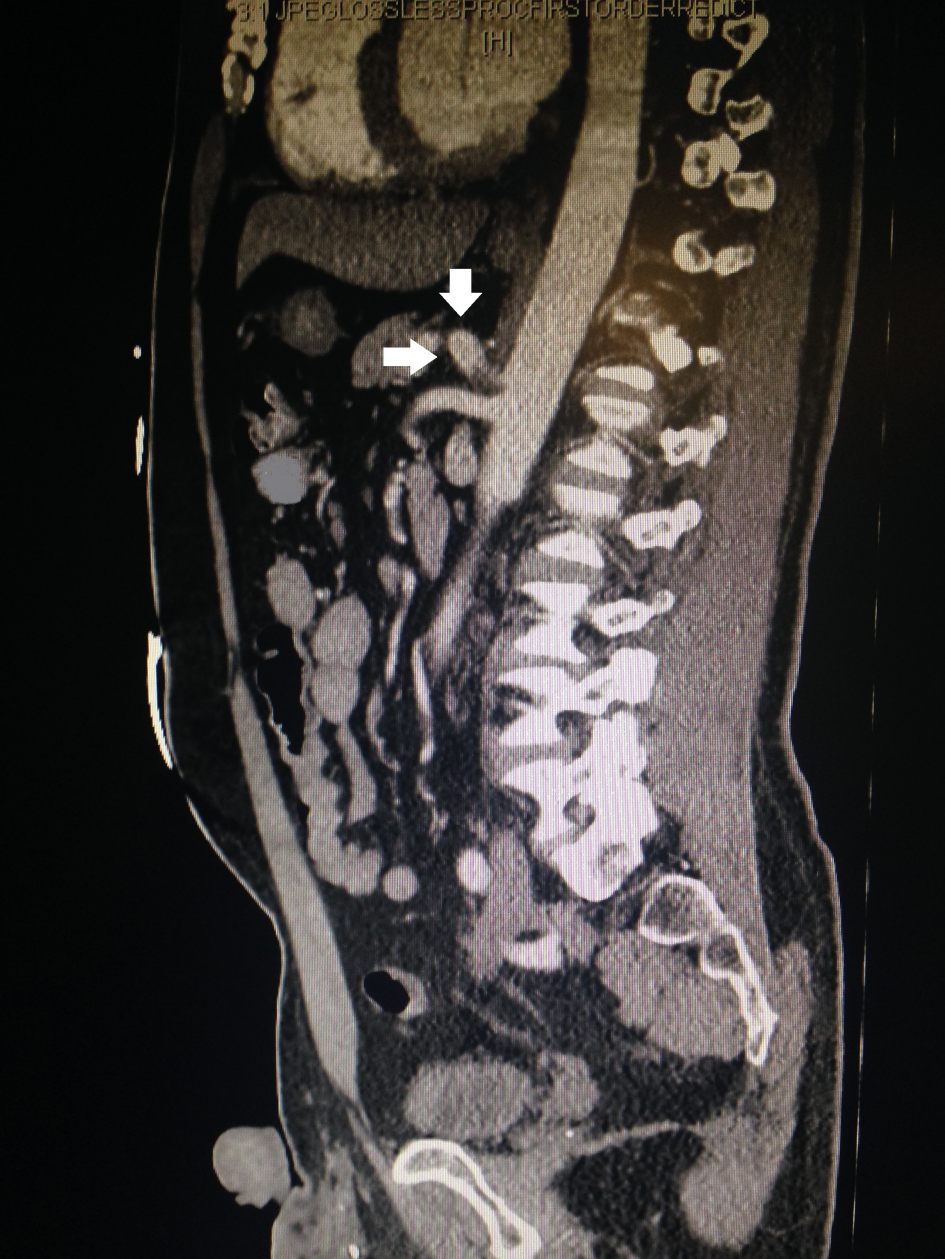
Computed tomography (CT) of abdomen and pelvis with contrast. Pseudoaneurysm involving the ostium of the celiac artery (arrows) with focal dissection.

A contrast-enhanced CT of abdomen and pelvis was ordered and showed a pseudoaneurysm involving the ostium of the celiac artery with focal dissection (Figure 1). There was no evidence of thrombosis or infarction to the liver, spleen, or bowel. Ultrasound studies of the abdominal aorta demonstrated a prominent celiac artery measuring 1.4 cm consistent with pseudoaneurysm; the abdominal aorta was normal with a patent celiac artery showing adequate distal flow (Video 1). Velocity at the celiac artery was minimally elevated at 142 cm/s and there was no evidence of a dynamic intimal flap. Due to these findings of isolated celiac artery dissection without evidence of further complications, the patient was managed conservatively with clopidogrel 75 mg and percocet 5 mg/325 mg and discharged after two days of observation. Follow-up repeat ultrasound three weeks following discharge showed no evidence of dissection flap, stenosis, or thrombosis and no increase in the size of the aneurysm. At this time, the patient was normotensive at 128/71 and reported no further episodes of abdominal pain. He was subsequently placed on aspirin 81 mg, with a plan to follow up as an outpatient annually at the vascular clinic.

**Video 1:** Ultrasound of abdominal aorta. Ultrasound study showing a normal abdominal aorta, a patent celiac artery with adequate distal blood flow, and no evidence of a dynamic intimal flap.

## Discussion

Spontaneous isolated celiac artery dissection in the absence of aortic dissection is an extremely rare disease and fewer than 100 cases have been reported in the last 14 years [1]. Isolated case reports in patients without compromised distal perfusion suggest that this condition can spontaneously resolve with medical therapy alone [1-3]; however, the extension of the dissection into adjacent vessels can lead to more serious complications including aneurysmal dilatation, complete occlusion, and rupture of a visceral artery [4]. Although certain risk factors such as hypertension, atherosclerotic disease, and connective tissue disorders have been linked to this condition, the exact etiology remains unclear [5-6]. Large prospective studies have not been conducted due to the rarity of this condition; therefore, optimal management has not been clearly defined and treatment primarily depends on clinical presentation and lesions identified on imaging studies.

Conservative medical treatment, endovascular procedures, and surgical revascularization are three possible options for managing spontaneous celiac artery dissections. Surgical and endovascular procedures are suggested when patients have persistent abdominal pain, organ ischemia, aneurysmal rupture, or continued propagation of the dissection [7]. Medical therapy often includes anticoagulation and/or antiplatelet therapy in conjunction with blood pressure control in order to prevent further thromboembolic complications [1]. However, there are currently no consistent recommendations regarding which patients should receive conservative medical therapy as opposed to surgical or endovascular procedures.

In 2014, Garrett led a systematic review of 88 cases on patients with isolated celiac dissection in attempt to delineate those who could be observed and those that required intervention [1]. In his study, patients had an average age of 53 years; 26 cases did not report treatment, 51 patients were initially treated with conservative medical therapy, and the remaining 11 required endovascular or surgical intervention. Six patients out of 51 failed initial medical management (one for aneurysmal evolution) and five out of 11 (45%) patients required endovascular or surgical management for ruptured aneurysms [8]. This study demonstrated conservative medical therapy was appropriate for most cases of celiac artery dissection in the absence of other serious complications; however, 45% of cases that necessitated endovascular or surgical repair were due to aneurysmal degeneration. Although a ruptured aneurysm would require emergent intervention, guidelines for elective treatment of a non-ruptured aneurysm have not yet been clearly established.

Due to the risk of rupture, most cases of reported celiac artery aneurysms follow treatment with endovascular repair or surgical bypass [9-10]. However, in another review, Oh, et al. reported six cases of isolated celiac dissection with aneurysms that were successfully treated with medical therapy alone except for one patient who received endovascular stent due to worsening abdominal pain. The remaining five patients were asymptomatic by 16 months following anticoagulation treatment and follow-up CT studies showed no progression of aneurysm size and preservation of distal perfusion [3]. Similarly, our patient’s abdominal pain resolved within three months following medical therapy, and the celiac artery aneurysm was stable at 1.4 cm. Conservative approach with antiplatelet and analgesic therapy was sufficient in treating our case of celiac artery dissection with pseudoaneurysm formation. We suggest endovascular or surgical intervention should be reserved for patients who present with hemodynamic instability, or other serious complications, such as aneurysm rupture or visceral infarction.

## Conclusions

Spontaneous isolated celiac artery dissection is a rare but potentially life threatening condition in the presence of pseudoaneurysm formation and rupture. At this time, there are no established guidelines for managing patients with this disease process. There is a general consensus among vascular surgeons that patients who present with no complications of celiac artery dissection may be managed conservatively with blood pressure control and antiplatelet or anticoagulation therapy. And on the other hand, it is generally agreed upon that patients who present with hemodynamic instability and evidence of severe complications such as ruptured aneurysms, malperfusion, and rapid propagation of the dissection would necessitate emergent endovascular or surgical intervention. However, management of patients with mild to moderate complications of celiac artery dissection, such as our patient with stable pseudoaneurysm formation, remains unclear. Although the risk of rupture for a visceral artery aneurysm has not yet been extensively studied, our patient had no evidence of end-organ ischemia or expansion of the celiac artery pseudoaneurysm; therefore we chose to initially treat our patient with conservative medical therapy along with close observation for aneurysmal dilatation and rupture. Our patient experienced no increase in the size of the aneurysm, suggesting that blood pressure control along with antiplatelet therapy is sufficient in managing patients with pseudoaneurysm formation from celiac artery dissection.
